# Effects of parasites coinfection with other pathogens on animal host: A literature review

**DOI:** 10.14202/vetworld.2022.2414-2424

**Published:** 2022-10-15

**Authors:** Wael M. Hananeh, Asya Radhi, Rami M. Mukbel, Zuhair Bani Ismail

**Affiliations:** 1 Department of Veterinary Pathology and Public Health, Faculty of Veterinary Medicine, Jordan University of Science and Technology, P.O. Box, 3030, Irbid 22110, Jordan; 2 Department of Basic Veterinary Medical Sciences, Faculty of Veterinary Medicine, Jordan University of Science and Technology, P.O. Box 3030, Irbid 22110, Jordan; 3 Department of Clinical Veterinary Medical Sciences, Faculty of Veterinary Medicine, Jordan University of Science and Technology, P.O. Box 3030, Irbid 22110, Jordan

**Keywords:** animals, coinfection, parasites

## Abstract

A parasite-host relationship is complicated and largely remained poorly understood, especially when mixed infections involving pathogenic bacteria and viruses are present in the same host. It has been found that most parasites are able to manipulate the host’s immune responses to evade or overcome its defense systems. Several mechanisms have been postulated that may explain this phenomenon in different animal species. Recent evidence suggests that coinfections involving many parasitic species alter the host’s vulnerability to other microorganisms, hinder diagnostic accuracy, and may negatively impact vaccination by altering the host’s immune responsiveness. The objective of this review was to provide a comprehensive summary of the current understanding of how parasites interact with other pathogens in different animal species. A better understanding of this complex relationship will aid in the improvement efforts of disease diagnosis, treatment, and control measures such as novel and effective vaccines and therapeutics for infectious diseases.

## Introduction

Parasites possess many mechanisms by which they manipulate or evade the immune response to establish infection [1]. Most studies regarding host-parasite relationships have been conducted in laboratory animals, primarily rodents, under a controlled environment [1]. Infection with multiple pathogens within the same host is common, as different infectious agents are obtained through similar routes of exposure (inhalation, oral, ingestion of contaminated feed, and water) [2]. The coinfecting agents interact with one another; their interaction may include direct and resource competition and immune-mediated interactions [3]. Each interaction can alter the dynamics of every single pathogen [4]. In the event of a coinfection, the way the two or more infectious agents interact can lead to different outcomes, including inhibition, activation, or competition between the coinfecting agents [5].

Coinfection between pathogens can affect susceptibility to other pathogens and, therefore, can alter host immune responsiveness [1]. In addition, coinfection can hinder accurate diagnosis and influence disease pathology development [6–10]. A previous study regarding parasites coinfection with other pathogens showed an apparent influence on disease susceptibility [10].

Therefore, the objective of this review was to summarize the complex relationships between parasites in a single host in different animal species.

## Literature Search

Data search was conducted using popular web search engines such as PubMed, Research Gate, and Google Scholar to extract all published papers related to the effects of parasites coinfection with other pathogens on host immunity in different animal species. Only published papers in scientific and refereed journals between the years 2000 and 2021 were considered. In total, 28 research papers published between 2000 and 2021 fit the inclusion criteria and were included in this analysis (Table-1) [11–38].

**Table-1:** Brief summary of published papers on the effects of parasites coinfection with other pathogens on host immunity in different animal species.

Title	Type of study and animal species	Brief summary	Reference
Effect of preexisting FeLV infection or FeLV and feline immunodeficiency virus coinfection on pathogenicity of the small variant of *H. felis* in cats	- Experimental - Cats	- Cats coinfected with FeLV and *H. felis* develop more critical anemia than cats infected with *H. felis* alone - Conclusion: *H. felis* induce myeloproliferative disease in cats infected with FeLV	[11]
Association of *F. gigantica* coinfection with bovine tuberculosis infection and diagnosis in a naturally infected cattle population in Africa	- Natural outbreak investigation - Cattle	- Coinfected cattle with *F. gigantic* and TB had a higher risk of developing TB lesions - *Fasciola gigantica* infection with bovine tuberculosis had lower IFN-γ levels - Conclusion: Breed-dependent differences in responses to coinfections with *F. gigantic* and TB were observed	[12]
Severity of bovine tuberculosis is associated with coinfection with common pathogens in wild boar	- Natural outbreak investigation - Wild boars	- Infection with PCV2, ADV, and *Metastrongylus* spp. positively correlated to tuberculosis severity in wild boars - Conclusion: Control measures such as vaccination against PCV2 and ADV and deworming of wild boars is recommended as part of tuberculosis control program in the wild boar	[13]
Trematode infections in pregnant ewes can predispose to mastitis during the subsequent lactation period	- Experimental - Sheep	- Sheep that have infection of liver flukes (*Dicrocoelium dendriticum* or *F. hepatica*) are more susceptible to mastitis in the immediate postpartum period - Conclusion: Trematode infection predisposes ewes to mastitis through increased blood concentrations of β-hydroxybutyrate which may adversely affect mammary cellular defenses	[14]
Chronic intestinal nematode infection exacerbates experimental *S. mansoni* infection	- Experimental - Mice	- Mice coinfected with *Trichuris muris* and *S. mansoni* showed significantly higher *S. mansoni* worm burdens and higher egg burden in the liver - Conclusion: *T. muris* induced alterations in lung cytokine expression and inflammatory foci surrounding lung stage schistosomula	[15]
Coinfection with the intestinal nematode *H. polygyrus* markedly reduces hepatic egg-induced immunopathology and pro-inflammatory cytokines in mouse models of severe schistosomiasis	- Experimental - Mice	- Mice infected with *H. polygyrus* have a marked reduction in schistosome egg-induced hepatic pathology - Conclusion: Intestinal nematodes prevent Th1- and Th17cell-mediated inflammation by promoting a strong Th2-polarized environment associated with increases in the levels of alternatively activated macrophages and T regulatory cells, which result in significant amelioration of schistosome-induced immunopathology	[16]
Coinfection with *S. mansoni* reactivates viremia in rhesus macaques with chronic simian-human immunodeficiency virus clade C infection	- Experimental - Rhesus macaques	- Coinfected monkeys with *S. mansoni* and chronic simian-human immunodeficiency virus clade C had significantly more fecal shedding of parasite eggs, eosinophilia, and increased viral replication - Conclusion: *S. mansoni* coinfection resulted in elevated mRNA expression of T-helper type 2 cytokine (Il-4) and induced T-cell subset alterations	[17]
Coinfection with *P. berghei* and *T. brucei* increases severity of malaria and trypanosomiasis in mice	- Experimental - Mice	- The severity of malaria increased when mice were coinfected with *P. berghei* and *T. brucei*. Mice with coinfection had lower survival rates, greater parasitemia loads, and more severe anemia	[18]
Trypanosoma infection favors Brucella elimination through IL-12/IFN-γ-dependent pathways	- Experimental - Mice	- Mice chronically infected with *B. melitensis*, *B. abortus*, or *B. suis* had lower bacterial loads in their spleen if they were coinfected with *T. brucei* - Conclusion: Strong pro-inflammatory IFN-γ-mediated response induced by *T. brucei* protects coinfected animals against Brucella	[19]
*Schistosoma mansoni*-*T. cruzi* coinfection modulates arginase-1/iNOS expression, liver and heart disease in mice	- Experimental - Mice	- Mice coinfected with *S. mansoni* and *T. cruzi* were unable to control *T. cruzi* infection with tremendous inflammation in their livers due to increased parasitemia. - Conclusion: *S. mansoni* reduced protection against *T. cruzi* due to reduced production of IFN-γ and NO	[20]
Experimental *T. gondii* and *E. tenella* coinfection in chickens	- Experimental - Chicken	- Coinfection of chickens with *T. gondii* and *E. tenella* did not show any detrimental effects on disease development or pathology	[21]
*Toxoplasma gondii* coinfection with diseases and parasites in wild rabbits in Scotland	- Natural outbreak investigation - Wild rabbits	- Wild rabbits coinfected with *T. gondii* and *E. stiedae* had higher burdens of *E. stiedae*	[22]
Toxoplasma coinfection prevents Th2 differentiation and leads to a helminth-specific Th1 response	- Experimental - Mice	- Coinfected mice with *H. polygyrus* and *T. gondii* displayed significantly higher worm fecundity - Conclusion: *T. gondii* infection limits a helminth-specific Th2 immune response while promoting a shift toward a Th1-type immune response	[23]
Enteric helminths promote *Salmonella* coinfection by altering the intestinal metabolome	- Experimental - Mice	- Mice coinfected with *H. polygyrus* and Salmonella showed enhanced pathogenesis of *S. enterica* serovar Typhimurium independently of actions of Th2 cells or regulatory T-cells - Conclusion: Infection with *H. polygyrus* disrupted the metabolic profile in the small intestine, thereby affecting the invasive capacity of *S.* Typhimurium	[24]
Generating super-shedders: Coinfection increases bacterial load and egg production of a gastrointestinal helminth	- Experimental - Mice	- Co-infected mice with *H. polygyrus* and *B. bronchiseptica* shed significantly more parasite eggs in their feces and had higher bacterial loads - Conclusion: Coinfection can be regarded as a mechanism that explains the often observed high variance in parasite load and shedding rates	[25]
Interactions between gastrointestinal parasitism and pneumonia in Nigerian goats	- Natural outbreak investigation - Goats	- Coinfected goats had pulmonary edema - Conclusion: Coinfection reduced the immunity in the lung, thereby allowing other pathogens (viruses and or bacteria) to create infection and facilitate the later development of pneumonia	[26]
Virus helminth coinfection reveals a microbiota-independent mechanism of immunomodulation	- Experimental - Mice	- Mice coinfected with *T. spiralis* or *H. polygyrus* and with mouse *Norovirus* (MNV) had increased viral loads and reduced amounts of specific CD4+ T cells expressing IFN-γ and TNF-alpha when compared to the mice infected with *Norovirus* alone - Conclusion: Parasite infection alters the immune response creating favorable environment for the parasite at the expense of antiviral immunity	[27]
Amelioration of influenza-induced pathology in mice by coinfection with *T. spiralis*	- Experimental - Mice	- Coinfection of mice during the enteric phase of trichinosis results in reduced lung pathology and accelerated recovery of weight - Conclusion: Infection with *T. spiralis* resulted in lower levels of tumor necrosis factor in bronchoalveolar lavage fluid and inhibited cellular recruitment into the airways of mice coinfected with influenza A virus	[28]
Nematode parasites and scrapie: Experiments in sheep and mice	- Experimental - Lambs, mice	- Lambs: Nematode infection shortened the development of scrapie with a significantly younger age at which first symptoms appeared - Mice: Parasitized mice demonstrated significant longer survival period - Conclusion: Nematodes modified the host susceptibility to scrapie	[29]
Coinfection with *F. hepatica* may increase the risk of *E. coli* O157 shedding in British cattle destined for the food chain	- Natural outbreak investigation - Cattle	- Coinfection with *F. hepatica* increases the risk of *E. coli* O157 fecal shedding. - Conclusion: Control of *F. hepatica* infection may have an impact on the shedding of *E. coli* O157 in cattle destined for the human food chain	[30]
Evaluation of the link between gyrodactylosis and streptococcosis of *Nile tilapia*, *O. niloticus* (L.)	- Experimental - Tilapia fish	- Co-infected fish with *S. iniae* and *G. niloticus* had increased mortality rates (42.2%) when compared to fish only infected with *S. iniae* (6.7%) - Conclusion: Gyrodactylus parasites carried viable bacterial cells, damaged fish epithelium, and allowed entry of the bacteria into the tissues	[31]
Enhanced mortality in *Nile tilapia* *O. niloticus* following coinfections with ichthyophthiriasis and streptococcosis	- Experimental - Tilapia fish	- Coinfected fish with *I. multifiliis* and *S. iniae* resulted in a negative impact on developmental size and an increase in mortality and parasite loads - Parasite load and trophont size increased susceptibility and mortality of tilapia to *S. iniae* infection - Conclusion: Coinfection permitted extra time for *I. multifiliis* to produce large, well-developed trophonts that allowed for more damage to epithelium of fish and thus more facilitated bacterial invasion, leading to higher mortality rates	[32]
*Dactylogyrus intermedius* parasitism enhances *F. columnare* invasion and alters immune-related gene expression in *C. auratus*	- Experimental - Goldfish	- Goldfish (*C. auratus*) infected with *D. intermedius* demonstrated increased susceptibility to *F. columnare.* - Confection resulted in higher mortality rates, and higher bacterial loads. - Conclusion: *D. intermedius* resulted in immunosuppression which enhanced bacterial invasion	[33]
Effect of *I. multifiliis* parasitism on the survival, hematology, and bacterial load in channel catfish previously exposed to *E. ictaluri*	- Experimental - Catfish	- Coinfection of catfish with *I. multifiliis* and *E. ictaluri* resulted in increased mortality rates and increased bacterial loads in different organs (71.1%) when compared to single infection groups - Conclusion: Coinfected catfish exhibited significant lymphopenia, suggesting that lymphocytes were actively involved in the immune response	[34]
*Ichthyophthirius multifiliis* as a potential vector of *E. ictaluri* in channel catfish	- Experimental - Catfish	- The study concluded that *I. multifiliis* could be a vector to *E. ictaluri*	[35]
Parasitism by protozoan *I. multifiliis* enhanced invasion of *Aeromonas hydrophila* in tissues of channel catfish	- Experimental - Catfish	- Coinfected catfish with *I. multifiliis* and *A. hydrophila* had increased mortality rate (80%), and higher amounts of *A. hydrophila* in their internal organs Conclusion: *I. multifiliis* infection leads to higher levels of cortisol in the channel catfish, leading to immune suppression	[36]
*Flavobacterium columnare*/*M. tilapiae* concurrent infection in the Earthen Pond Reared Nile Tilapia (*O. niloticus*) during the early summer	- Natural outbreak investigation - *Nile tilapia*	- Coinfection of tilapia fish with *F. columnare* and *M. tilapiae* resulted in high mortality rate - *F. columnare* and *M. tilapiae* exhibited a synergistic effect to induce severe pathology resulting in fish mass mortalities - Conclusion: Damage to the fish skin by *M. tilapiae* may have allowed for more *F. columnare* invasion in tissues	[37]
Immunomodulation and disease resistance in post-yearling rainbow trout infected with *M. cerebralis*, the causative agent of whirling disease	- Experimental - Rainbow trouts	- Rainbow trouts were chronically infected with *M. cerebralis* and challenged with *Y. ruckeri* - Coinfected rainbow trouts had higher mortality rates and faster onset of death than in rainbow trout without *M. cerebralis* infection - Conclusion: *M. cerebralis* modulate the immune response by inducing leukocyte suppression	[38]

*H. felis=Haemobartonella felis, F. gigantica=Fasciola gigantica, S. mansoni=Schistosoma mansoni, H. polygyrus= Heligmosomoides polygyrus, S. Typhimurium=Salmonella Typhimurium, S. enterica=Salmonella enterica, P. berghei=Plasmodium berghei, T. brucei=Trypanosoma brucei, M. cerebralis=Myxobolus cerebralis, B. melitensis=Brucella melitensis, B. abortus=Brucella abortus, or B. suis=Brucella suis, O. niloticus=Oreochromis niloticus, F. columnare=Flavobacterium columnare, M. tilapiae=Myxobolus tilapiae, I. multifiliis=Ichthyophthirius multifiliis, E. ictaluri=Edwardsiella ictaluri, T. cruzi=Trypanosoma cruzi, T. gondii=Toxoplasma gondii, E. tenella=Eimeria tenella, T. spiralis=Trichinella spiralis, F. hepatica=Fasciola hepatica, E. coli=Escherichia coli, D. intermedius=Dyschirius intermedius, C. auratus=Carassius auratus,* PCV2=Porcine circovirus type 2, ADV=Aujeszky’s disease virus, FeLV=Feline leukemia virus, IFN-γ=Interferon-gamma, IL-12=Interleukin-12, NO=Nitrous oxide

## Mechanisms of Parasites-pathogens Interactions within a Single Host (Coinfection)

The net result of multiple infecting pathogens within a single host could be characterized as synergistic (intensifying adverse effects of individual pathogens) or antagonistic (lessening of adverse effects of individual pathogens) [39]. The potential mechanisms of this interaction remain speculative and many theories have been suggested to explain this complex relationship. Among the most widely accepted theories are the community ecology theory (competitive interactions between pathogens sharing similar resources or location in a host) and pathogen-host immune modification theory, where a pathogen modifies the host immune system and a new infecting parasite or pathogen finds an altered immunoenvironment created in response to previous or current infections [39]. The antagonistic effect of coinfecting pathogens has been suggested to occur when one pathogen inhibits the invasion, development, or reproduction of another pathogen within the host [40]. Within this context, certain species of nematodes and bacteria could block the transmission of some trematodes by biologically controlling intermediate hosts and relieving symptoms by suppressing infections by other pathogens, including viruses, bacteria, and parasites [40]. Parasites, especially helminths, are known to produce a robust Th2-type response which can downregulate the effects of a secondary T-helper (Th) 1-dependent parasitic challenge [40]. This typically results in a rapid proliferation of secondary pathogens due to T-cell inhibition. Furthermore, severe immunopathological damage could ensue due to uncontrollable inflammatory reactions [40].

On the other hand, strong clinical and scientific evidence exists that suggest that certain parasite infections may protect the host by reducing the density of pathogens and mitigating immunopathological injury [40]. It was found that infection with certain species of parasites may dampen or inhibit the maturation of dendritic cells (DC) and promote the propagation of regulatory T (Treg) cells [40]. Several studies have indicated that DCs induce an adaptive immune response in the early phases of parasite infections [40]. In addition, some parasites produce toxic compounds that directly eliminate or hinder the development of pathogenic competitors within the host [40]. Interference competition can also occur when certain cestodes produce some substances termed crowding factors [40]. This results when end metabolites accumulate in the microenvironment of the host, acting as a growth inhibitor of competing pathogens [40].

## Examples of Parasite-pathogen Coinfection Interactions

### Bovine tuberculosis (TB) and *Fasciola hepatica* coinfection

Bovine TB, caused by *Mycobacterium bovis*, is a zoonotic disease and is considered one of the most important diseases of cattle throughout the world. At present, diagnosis of TB in cattle is based on the single intradermal comparative cervical tuberculin test and the *in vitro* interferon-gamma (IFN-γ) assay [41].

In England and Wales, an epidemiological study was conducted on bovine dairy herds [12]. It was found that as the incidence of *F. hepatica* increased, more animals were detected as TB negative reactors [11]. Similar findings were obtained from a naturally concurrent *F. gigantica* infection with bovine TB in African cattle [42]. Meanwhile, experiments performed in bovine herds confirmed that bovine TB coinfection with *F. hepatica* affects the sensitivity of bovine TB tests by reducing the host Th1 immune responses against bovine TB [43]. In a recent review, it was concluded that liver fluke infection may affect the diagnosis of bovine TB [44].

### Bovine TB and gastrointestinal nematodes

There are many studies that addressed the coinfection relationship between gastrointestinal nematodes and bovine TB [1, 45, 46]. A compilation of experimental, cross-sectional, and modeling approaches has been utilized to study the nature of this coinfection relationship. Most of these studies reported that nematode infection decreases host’s Th1 response which may result in an increased parasite susceptibility [13,47]. It was also found that animals infected with TB showed less severe coinfection with nematodes. This relationship was correlated with weakened Th1 responses against TB antigen. These results clearly indicate that TB-infected animals may express stronger anti-nematode defenses associated with lessened control over the concurrent TB infection [47]. It was also concluded that TB coinfected animals may develop a rapid onset, leading to increased infectiousness potential [13]. A study conducted in Portugal explores the effects of coinfection of wild boars with TB and *Metastrongylus* spp. (Swine lungworm), a direct and strong correlation was found between the severity of TB and *Metastrongylus* spp. coinfection [13].

### Human TB

*Mycobacterium tuberculosis* is the causative agent for human TB, a chronic disease affecting one-third of the human population worldwide [48, 49]. The bacterium mainly targets the lung with most of the infected individuals are asymptomatic with latent disease. It was noted that TB coinfected individuals with parasites often had more severe disease [48, 49]. The previous studies [48, 49] of TB coinfection with parasitic diseases in humans have shown significant modulation of host’s immune responses in which coinfected people suffered more severe symptoms of TB. It has been shown that coinfection with leishmaniasis, lepromatous leprosy, and TB downregulated the Th1 cell response significantly [49]. Coinfection with TB and malaria was also reported to decrease or suppress immune responses against TB, resulting in a more protracted and chronic course of TB in infected people [49]. These results may suggest a competitive antagonist effect TB and malaria parasites. Furthermore, coinfection between TB and echinococcosis was reported to increase the chronicity of TB infection in which the immune responses were changed from a Th1 to Th2 response [49].

## Trematodes and Pregnancy Toxemia in Sheep

In the advanced stages of pregnancy in sheep, pregnancy toxemia occurs due to abnormal carbohydrate metabolism. Pregnancy toxemia is characterized by increased blood ketone bodies. One of these ketone bodies (B-hydroxybutyrate) is used as an indicator that can assess the development of pregnancy toxemia in sheep. It was reported that increased hyperketonemia with such a ketone body was present in trematode-infected ewes more than in their non-infected ewes [14]. Mavrogianni *et al*. [14] hypothesized that B-hydroxybutyrate could modulate the immune response by which the ketone bodies negatively impact the immunity in the udder. Therefore, the udder would be at risk of infection. The same authors postulated that the use of anthelmintic drugs could decrease the incidence of udder infection in sheep [14].

## Blood Flukes (Schistosomiasis)

Schistosomes or blood flukes in humans and animals could cause chronic inflammation [1]. Similar to other parasites, schistosomes induce a Th2 immune response, which allows them to induce a long-lasting infection with the host [1]. It was demonstrated that gastrointestinal nematode infections in mice could have an impact on the pathogenesis of schistosome infection in the host [15]. Mice with an established *Trichuris muris* infection had enhanced survival and migration of *Schistosome mansoni* infection [15]. In another mice study, it was shown that differences in the coinfecting species can have variable effects on schistosome infections [16]. *Heligmosomoides polygyrus* specifically induces infection in the duodenum of murine species. It was reported that coinfection of mice with *Schistosome* spp. and *H. polygyrus* infections ameliorated the hepatic pathology induced by schistosomes’ eggs [16]. The phenomenon seemed to correlate with the decreased expression of pro-inflammatory cytokines [16]. Therefore, the above-mentioned studies demonstrated that gastrointestinal helminths may impact schistosomiasis. However, these data do not predict whether they increase or decrease disease pathogenesis. Some experimental work suggested that the large intestinal mucosa allowed the gastrointestinal helminth to initiate chronic infections [50, 51]. This phenomenon can increase the host’s susceptibility to other infections with helminth parasites [46]. It was reported that African buffalo positive for *Cooperia fuelleborni* nematode exhibited increased S*chistosoma mattheei* burden than those animals negative for the nematode species [46]. Similar results were seen in cattle infected with *Cooperia oncophora* by which the infection enhanced their susceptibility to lungworms (*Dictyocaulus viviparus*) infection [51]. Furthermore, it was shown that calves infected with *Ostertagia ostertagi* expressed an increased susceptibility to lungworm infestation [51]. The mechanisms that mediate these effects in coinfected animals are still not understood and could just represent variability in different gastrointestinal helminthic infections [1].

An experimental study was done in *Rhesus macaques* to examine if helminth parasite coinfection would create higher viremia loads of simian-human immunodeficiency virus (SHIV) clade C [17]. In that study, a group of monkeys was coinfected with *S. mansoni* cercariae and SHIV. By week 5 after infection, coinfected monkeys were reported to significantly shed more eggs in their feces, had eosinophilia, and increased mRNA expression of Th type 2 cytokine ([Il]-4) than monkeys without schistosomiasis infection [17]. Coupled with that, viral replication was profoundly increased in coinfected monkeys compared to controls. Therefore, *S. mansoni* coinfection resulted in increased viral replication and induced T-cell subset alternations in monkeys with chronic SHIV clade C infection [17].

## Trypanosomes

In an experimental study, it was reported that the severity of malaria was increased when mice were coinfected with *Plasmodium berghei* and *Trypanosoma brucei* [18]. The mice with the coinfection had lower survival rates, greater parasitemia loads, and severe anemia. The authors were uncertain about the mechanism of such effects on the increased expression of IFN-γ in the coinfected mice [18]. In addition, *trypanosome* infection was also found to ameliorate the vulnerability of the host to bacterial infections [19]. In a study conducted in chronically infected mice with either *Brucella melitensis*, *B. abortus*, or *B. suis*, it was noted that bacterial loads in the spleen were decreased when they coinfected with *T. brucei* [19]. The authors demonstrated that coinfection with *T. brucei* increased the immune control of chronic Brucella infection and eliminated infection by enhancing CD4+ T cells dependent Th1 immune response [19]. On the other hand, *T. brucei* infection failed to induce similar effects as those seen in *Brucella* spp. infections study in mice when coinfected with *Mycobacterium tuberculosis* [1, 19]. In another study conducted in mice, it was found that there was a significant alteration in the mechanism of disease as well as the susceptibility of mice to *T. cruzi* infection that was previously infected with *S. mansoni* [20]. The mice were unable to control *T. cruzi* infection, and there was tremendous inflammation in their livers due to increased parasitemia [20]. *T. cruzi* infection was found to be associated with Th1 immune response and the production of nitrous oxide (NO) which is needed to clear the parasite [20]. Therefore, increased parasitemia and its associated inflammation were seen due to reduced production of IFN-γ and NO.

## *Eimeria* spp. and *Toxoplasma gondii* Coinfection

*Eimeria* spp. is a genus of apicomplexan parasites that lead to the disease coccidiosis in a variety of animal species, including cats, dogs, poultry, and ruminants. *Eimeria tenella* is a notable pathogen that causes intestinal coccidiosis in chickens and causes high morbidity and mortality. Toxoplasmosis is caused by *T. gondii* infection. The disease is an important cause of reproductive failure and abortion in animals with significant zoonotic potential. In normal animals, toxoplasmosis is frequently subclinical and chronic in immunocompetent individuals. In poultry, it is common to have *E. tenella* and *T. gondii* coinfection [21]. In different experimental studies that addressed the coinfection relationship between *E. tenella* and *T. gondii*, it was noted that the immunopathology responses following coinfection had no differences from those chickens infected with *Eimeria* alone [1, 21, 22]. Moreover, the coinfection showed no effect on the burden of tissue samples with *T. gondii* or its clinical course [1, 21, 22]. Therefore, those coinfections in chickens did not show any detrimental effects on disease development or pathology [1, 21, 22]. On the contrary, a study conducted on wild rabbits in Scotland showed that rabbits with coinfection of *T. gondii* and *E. stiedae* had higher burdens of *E. stiedae* [22].

## *Toxoplasma gondii* and *H. polygyrus* Coinfection

A coinfection study was performed to study the immunological relationship between the enteric nematode *H. polygyrus* in mice with a previous infection of *T. gondii* [23]. It was found that *T. gondii* parasitemia was not affected by concurrent helminth infection [23].

## *Salmonella* spp.

Salmonella is a Gram-negative bacterium and is an important cause of diarrhea worldwide. Salmonella coinfection with different parasites has been shown to aggravate the disease process of salmonellosis [1]. An experimental study of mice coinfected with *H. polygyrus* and *Salmonella* demonstrated that *H. polygyrus* infection had enhanced the pathogenesis of *Salmonella enterica* serovar Typhimurium [24]. It was shown that infection with *H. polygyrus* can disrupt metabolic profile in the small intestine, thereby affecting the invasiveness of *Salmonella* Typhimurium [24]. The parasite appeared to enhance the expression of *Salmonella* pathogenicity island 1 genes [24]. The study provided awareness of how parasites could modulate the susceptibility of the host to other bacterial infections [24].

## *Bordetella bronchiseptica* and *H. polygyrus* Coinfection

In an experimental study conducted to study the impact of coinfection of *H. polygyrus* and *B. bronchiseptica* in laboratory mice, it was found that coinfection led to the presence of higher bacterial loads early in the infection (first 5 days) that could lead to host mortality [25]. Coupled with that, coinfection led to the development of “super-shedders,” which are individuals that will shed helminth eggs chronically with larger than average amounts [25]. Lass *et al*. [25] proposed that coinfection can be an underlying factor in how parasites can have inconsistency in their parasitic load and shedding rates, and this should be taken into consideration for the control and management of the disease [25].

## Pneumonia and Gastrointestinal Parasitism

A study was done on goats in Nigeria to investigate if there was any relationship between occurrence of pneumonia and gastrointestinal parasitism [26]. Based on the data obtained in the study, a significant correlation was present between the presence of gastrointestinal parasitism and granulomatous pneumonia. The authors suggested that gastrointestinal parasites could have decreased the immunity in the lung, thereby allowing other pathogens to invade the lungs resulting in pneumonia [26].

## Viruses and Parasites Coinfection

There are some studies exploring the coinfection relationship between parasites and viruses [27, 28]. In those scenarios, a Th2 immune response from the parasites impeded the development of Th1 immune response from the viral pathogen, thereby hindering effective antiviral immunity. An experimental study revealed that mice coinfected with *Trichinella spiralis* or *H. polygyrus* and with *Mouse norovirus* had increased viral loads and reduced amounts of specific CD4+ T cells expressing IFN-γ and TNF-α when compared to the mice infected with *Norovirus* alone [27]. Another experimental study showed that mice infected with *H. polygyrus* or *S. mansoni* could encourage the reactivation of latent γ-herpesvirus infection [27].

Another experimental study demonstrated that coinfection of *T. spiralis* led to reduced levels of human necrosis factor in bronchoalveolar lavage fluid and inhibited cellular recruitment into the airways of mice coinfected with influence A virus [28]. The authors stated that *T. spiralis* ameliorated the pulmonary pathology, causing less CD4+ and CD8+ lymphocytes and neutrophils recruitment and suppressed the virus from the increasing effect of vascular permeability in pulmonary tissue [28].

## Scrapie and *Teladorsagia circumcincta* Coinfection

In an experimental study conducted to explore the impact of abomasal parasite, *T. circumcincta* infection, on the host susceptibility to scrapie using sheep naturally exposed to scrapie, chosen by their genotype at the *PrP* gene, it was found that the onset of prion disease was shortened in coinfected sheep [29].

## *Escherichia coli* (O157)

*Escherichia coli* O157 is an important zoonotic bacterium of worldwide public health significance. It causes hemorrhagic diarrhea in infected humans. The main reservoir for human infection is cattle, and the animals are generally asymptomatic. A study was performed in 2018; on 14 British farms showed that coinfection with the liver fluke (*F. hepatica*) might increase the risk of *E. coli* (O157) shedding [30]. The study proposed that controlling *F. hepatica* in cattle could have a beneficial effect by lowering the shedding of *E. coli* (O157) [1, 30].

## Feline Leukemia Virus (FeLV) and Feline Immunodeficiency Virus (FIV) Coinfection with *Mycoplasma haemofelis* Formerly (*Haemobartonella felis*)

Feline infectious anemia is caused by an epicellular bacterial parasite of the erythrocytes of cats that can cause hemolytic anemia. The causative agent is *M. haemofelis* (formerly *H. felis*). A study was conducted to explore the effects of a pre-existing FeLV infection or FIV on the pathogenicity of *M. haemofelis*. The results of the experimental study showed that cats coinfected with FeLV and *M. haemofelis* developed more critical anemia than cats infected with *M. haemofelis* alone. It was, however, unclear how the two infectious agents acted together to produce a more severe disease [11].

## Coinfections in Fish

Many experimental studies displayed the synergistic relationship between various parasitic spp. and bacterial infections in fish. Parasitic infections are thought to increase to decrease the disease resistance of fish to other secondary bacterial infections. In addition, certain parasites may harbor pathogenic bacteria that infect the host while the parasites are feeding [5]. Mixed infections are common in fish, leading to increased morbidity and mortality. On the other hand, most studies focused on the dynamics of a single pathogen. A coinfection experimental study was done on *Nile tilapia* [31]. The fish in the study were infected with *Gyrodactylus niloticus* (monogenean helminthic ectoparasite) and then were later infected with *Streptococcus iniae* bacteria. The study demonstrated that increased mortality rates were observed in coinfected group (42.2%) when compared to fish infected with *S. iniae* only group (6.7%). No deaths were reported in the fish only infected with *G. niloticus* group. A study conducted by Xu *et al*. [31] proposed the ectoparasite facilitated the entry of pathogenic bacteria through mechanical damage to fish epithelium [31]. Xu *et al*. [32] conducted an experimental model of coinfection in *N. tilapia*. *Nile tilapia* had a coinfection of *I. multifiliis* and *S. iniae*, and the coinfection relationship had a negative impact on developmental size. There was an increase in mortality and parasite loads. When the time between exposures was increased, the coinfection permitted extra time for *I. multifiliis* to produce large, well-developed trophonts that allowed for more damage to the epithelium of fish and thus more bacterial invasion.

Another study conducted on Goldfish (*Carassius auratus*) demonstrated that goldfish with *Dactylogyrus intermedius* infection demonstrated increased susceptibility to *Flavobacterium columnare* bacteria [33]. The coinfection interaction led to higher mortality rates, and higher bacterial loads compared to fish infected with *F. columnare* alone. The parasite monogenean *D. intermedius* resulted in enhanced bacterial invasion after induction of host immune suppression. There was downregulation of immune genes such as TGF-B and complement 3 in gills and kidneys. Thus, *D. intermedius* modulates host’s immune response [33].

Shoemaker *et al*. [34] performed an experimental study to investigate the effects of *Ichthyophthirius multifiliis* (ciliated ectoparasite fish protozoan) parasitism on survival and bacterial burden of channel catfish exposed 1 day before to *Edwardsiella ictaluri* (pathogen causes enteric septicemia of catfish). The coinfected group of catfish had increased mortality rates and increased bacterial loads in different organs (71.1%) when compared to single infection groups [34]. Xu *et al*. [35] executed another experiment using *I. multifiliis* and *E. ictaluri* in channel catfish. First, the fish were infected with *I. multifiliis*, and then, the fish were challenged 5 days later with *E. ictaluri*. There were increased mortality and higher bacterial burden in internal organs, and the results were similar to the previous studies [5, 36]. High mortality rates were observed in *N. tilapia* in the 2009 summer season in Egypt. The significant high mortality rates were the result of *F. columnare* and myxosporean parasite, *Myxobolus tilapiae* coinfection. Damage to the fish skin may have allowed for more *F. columnare* invasion [37].

A study was done on rainbow trout to investigate if coinfection of *Myxobolus cerebralis* and *Yersinia ruckeri* resulted in higher mortality rates [38]. Rainbow trout in the study was chronically infected with *M. cerebralis* and 12 months post-exposure were challenged with *Y. ruckeri*. The coinfected rainbow trout had higher mortality rates and the onset of death was faster than in rainbow trout without *M. cerebralis* infection. Authors in the study suggested that *M. cerebralis* has immunomodulatory effects such as suppression of lymphocytes to four mitogens. This, therefore, allowed for a more destructive invasion by secondary infection with *Y. ruckeri* [38].

## Conclusion

It is common to have two or more pathogens coexisting in a single host. The host-pathogen relationship is complex and remains largely unexplored. This review highlighted the impact of parasites on the susceptibility or disease pathogenesis of coinfecting agents in different hosts. Some studies demonstrated that a parasitic coinfection exacerbated disease and led to rapid or more severe onset of disease, while other studies showed that coinfection status could lead to increased shedding of infectious agents. In addition, some studies showed that coinfection could alter the sensitivity of some diagnostic tests for a particular disease. A few studies determined that a coinfection of bacterial pathogen and parasite could lead to the reduction of parasite egg-induced pathology, and the phenomena correlated with the expression of pro-inflammatory cytokines responsible for induction of egg-induced immunopathology. A better understanding of how infectious disease pathogenesis can be influenced by concurrent parasite infections will improve or revolutionize existing diagnostic and control strategies for infectious diseases. However, further studies are still required to explore the nature and immunological interactions between coinfecting agents in a single host.

## Authors’ Contributions

WMH: Conceived the study. AR: Performed literature search and drafted the manuscript. RMM: Reviewed and edited the manuscript. ZBI: Performed final manuscript revision. All authors have read and approved the final manuscript.

## References

[1] Mabbott N.A (2018). The influence of parasite infections on host immunity to coinfection with other pathogens. Front. Immunol.

[2] Bosch J, Monsalve-Carcaño C, Price S.J, Bielby J (2020). Single infection with *Batrachochytrium dendrobatidis* or *Ranavirus* does not increase the probability of coinfection in a montane community of amphibians. Sci. Rep.

[3] Ezenwa V.O (2021). Coinfection and nutrition:Integrating ecological and epidemiological perspectives. Nutrition and Infectious Diseases.

[4] Nickbakhsh S, Mair C, Matthews L, Reeve R, Johnson P.C.D, Thorburn F, von Wissmann B, Reynolds A, McMenamin J, Gunson R.N, Murcia P.R (2019). Virus-virus interactions impact the population dynamics of influenza and the common cold. Proc. Natl. Acad. Sci. U. S. A.

[5] Kotob M.H, Menanteau-Ledouble S, Kumar G, Abdelzaher M, El-Matbouli M (2017). The impact of coinfections on fish:A review. Vet. Res.

[6] Viney M.E, Graham A.L (2013). Patterns and processes in parasite coinfection. Adv. Parasitol.

[7] Babu S, Nutman T.B (2019). Immune responses to helminth infection. Clinical Immunology.

[8] Lello J, McClure S.J, Tyrrell K, Viney M.E (2018). Predicting the effects of parasite coinfection across species boundaries. Proc. Biol. Sci.

[9] Nutman T.B (2015). Looking beyond the induction of Th2 responses to explain immunomodulation by helminths. Parasite Immunol.

[10] Cutler S.J, Vayssier-Taussat M, Estrada-Peña A, Potkonjak A, Mihalca A.D, Zeller H (2021). Tick-borne diseases and coinfection:Current considerations. Ticks Tick Borne Dis.

[11] George J.W, Rideout B.A, Griffey S.M, Pedersen N.C (2002). Effect of preexisting FeLV infection or FeLV and feline immunodeficiency virus coinfection on pathogenicity of the small variant of *Haemobartonella felis* in cats. Am. J. Vet. Res.

[12] Kelly R.F, Callaby R, Egbe N.F, Williams D.J, Victor N.N, Tanya V.N, Sander M, Ndip L, Ngandolo R, Morgan K.L, Handel I.G (2018). Association of *Fasciola gigantica* co-infection with bovine tuberculosis infection and diagnosis in a naturally infected cattle population in Africa. Front. Vet. Sci.

[13] Risco D, Serrano E, Fernández-Llario P, Cuesta J.M, Gonçalves P, García-Jiménez W.L, Martínez R, Cerrato R, Velarde R, Gómez L, Segalés J (2014). Severity of bovine tuberculosis is associated with coinfection with common pathogens in wild boar. PLoS. One.

[14] Mavrogianni V.S, Papadopoulos E, Spanos S.A, Mitsoura A, Ptochos S, Gougoulis D.A, Barbagianni M.S, Kyriazakis I, Fthenakis G.C (2014). Trematode infections in pregnant ewes can predispose to mastitis during the subsequent lactation period. Res. Vet. Sci.

[15] Bickle Q.D, Solum J, Helmby H (2008). Chronic intestinal nematode infection exacerbates experimental *Schistosoma mansoni* infection. Infect. Immun.

[16] Bazzone L.E, Smith P.M, Rutitzky L.I, Shainheit M.G, Urban J.F, Setiawan T, Blum A.M, Weinstock J.V, Stadecker M.J (2008). Coinfection with the intestinal nematode *Heligmosomoides polygyrus* markedly reduces hepatic egg-induced immunopathology and pro-inflammatory cytokines in mouse models of severe schistosomiasis. Infect. Immun.

[17] Ayash-Rashkovsky M, Chenine A.L, Steele L.N, Lee S.J, Song R, Ong H, Rasmussen R.A, Hofmann-Lehmann R, Else J.G, Augostini P, McClure H.M (2007). Coinfection with *Schistosoma mansoni* reactivates viremia in rhesus macaques with chronic simian-human immunodeficiency Virus Clade C infection. Infect. Immun.

[18] Ademola I.O, Odeniran P.O (2016). Co-infection with *Plasmodium berghei* and *Trypanosoma brucei* increases severity of malaria and trypanosomiasis in mice. Acta Trop.

[19] Machelart A, van Vyve M, Potemberg G, Demars A, de Trez C, Tima H.G, Vanwalleghem G, Romano M, Truyens C, Letesson J.J, Muraille E (2017). Trypanosoma infection favors *Brucella* elimination via IL-12/IFNg-dependent pathways. Front. Immunol.

[20] Rodrigues J.P.F, Caldas I.S, Gonçalves R.V, Almeida L.A, Souza R.L.M, Novaes R.D (2017). *S. mansoni*-*T. cruzi* co-infection modulates arginase-1/iNOS expression, liver and heart disease in mice. Nitric Oxide.

[21] Hiob L, Koethe M, Schares G, Goroll T, Daugschies A, Bangoura B (2017). Experimental *Toxoplasma gondii* and *Eimeria tenella* coinfection in chickens. Parasitol. Res.

[22] Mason S, Dubey J.P, Smith J.E, Boag B (2015). *Toxoplasma gondii* coinfection with diseases and parasites in wild rabbits in Scotland. Parasitology.

[23] Ahmed N, French T, Rausch S, Kühl A, Hemminger K, Dunay I.R, Steinfelder S, Hartmann S (2017). Toxoplasma coinfection prevents Th2 differentiation and leads to a helminth-specific Th1 response. Front. Cell. Infect. Microbiol.

[24] Reynolds L.A, Redpath S.A, Yurist-Doutsch S, Gill N, Brown E.M, van Der Heijden J, Brosschot T.P, Han J, Marshall N.C, Woodward S.E, Valdez Y (2017). Enteric helminths promote *Salmonella* coinfection by altering the intestinal metabolome. J. Infect. Dis.

[25] Lass S, Hudson P.J, Thakar J, Saric J, Harvill E, Albert R, Perkins S.E (2013). Generating super-shedders:Co-infection increases bacterial load and egg production of a gastrointestinal helminth. J. R. Soc. Interface.

[26] Adeyemi M.T, Morenikeji O.A, Emikpe B.O, Jarikre T.A (2017). Interactions between gastrointestinal parasitism and pneumonia in Nigerian goats. J. Parasit. Dis.

[27] Osborne L.C, Monticelli L.A, Nice T.J, Sutherland T.E, Siracusa M.C, Hepworth M.R, Tomov V.T, Kobuley D, Tran S.V, Bittinger K, Bailey A.G (2014). Virus-helminth coinfection reveals a microbiota-independent mechanism of immunomodulation. Science.

[28] Furze R.C, Hussell T, Selkirk M.E (2006). Amelioration of influenza-induced pathology in mice by coinfection with *Trichinella spiralis*. Infect. Immun.

[29] Gruner L, Elsen J.M, Vu Tien Khang J, Eychenne F, Caritez J.C, Jacquiet P, Andreoletti O, Sarradin P, Cortet J, Richer N, Leroux H (2004). Nematode parasites and scrapie:Experiments in sheep and mice. Parasitol. Res.

[30] Howell A.K, Tongue S.C, Currie C, Evans J, Williams D.J, McNeilly T.N (2018). Co-infection with *Fasciola hepatica* may increase the risk of *Escherichia coli O157* shedding in British cattle destined for the food chain. Prev. Vet. Med.

[31] Xu D.H, Shoemaker C.A, Klesius P.H (2007). Evaluation of the link between gyrodactylosis and streptococcosis of *Nile tilapia* and *Oreochromis niloticus* (L.). J. Fish Dis.

[32] Xu D.H, Shoemaker C.A, Klesius P.H (2009). Enhanced mortality in Nile tilapia *Oreochromis niloticus* following coinfections with ichthyophthiriasis and streptococcosis. Dis. Aquat. Org.

[33] Zhang C, Li D.L, Chi C, Ling F, Wang G.X (2015). *Dactylogyrus* intermedius parasitism enhances *Flavobacterium columnare* invasion and alters immune-related gene expression in *Carassius auratus*. Dis. Aquat. Organ.

[34] Shoemaker C.A, Martins M.L, Xu D.H, Klesius P.H (2012). Effect of *Ichthyophthirius multifiliis* parasitism on the survival, hematology and bacterial load in channel catfish previously exposed to *Edwardsiella ictaluri*. Parasitol. Res.

[35] Xu D.H, Shoemaker C.A, Klesius P.H (2012). Ichthyophthirius multifiliis as a potential vector of *Edwardsiella ictaluri* in channel catfish. FEMS. Microbiol. Let.

[36] Xu D.H, Pridgeon J.W, Klesius P.H, Shoemaker C.A (2012). Parasitism by protozoan *Ichthyophthirius multifiliis* enhanced invasion of *Aeromonas hydrophila* in tissues of channel catfish. Vet. Parasitol.

[37] Eissa A.E, Zaki M.M, Aziz A.A (2010). *Flavobacterium columnar*e*/Myxobolus tilapia*e concurrent infection in the earthen pond reared *Nile tilapi*a *(Oreochromis niloticu*s) during the early summer. Interdisc. Bio Centr.

[38] Densmore C.L, Ottinger C.A, Blazer V.S, Iwanowicz L.R, Smith D.R (2004). Immunomodulation and disease resistance in post-yearling rainbow trout infected with *Myxobolus cerebra is*, the causative agent of whirling disease. J. Aquat. Anim. Health.

[39] Thumbi S.M, de C Bronsvoort B.M, Poole E.J, Kiara H, Toye P, Ndila M, Conradie I, Jennings A, Handel I.G, Coetzer J.A, Hanotte O, Woolhouse M.E (2013). Parasite coinfections show synergistic and antagonistic interactions on growth performance of East African zebu cattle under one year. Parasitology.

[40] Shen S.S, Qu X.Y, Zhang W.Z, Li J, Lv Z.V (2019). Infection against infection:Parasite antagonism against parasites, viruses and *Bacteria*. Infect. Dis. Poverty.

[41] Garza?Cuartero L, O'sullivan J, Blanco A, McNair J, Welsh M, Flynn R.J, Williams D, Diggle P, Cassidy J, Mulcahy G (2016). *Fasciola hepatica* infection reduces *Mycobacterium bovis* burden and mycobacterial uptake and suppresses the pro?inflammatory response. Parasite Immunol.

[42] Flynn R.J, Mulcahy G, Welsh M, Cassidy J.P, Corbett D, Milligan C, Andersen P, Strain S, McNair J (2009). Co?infection of cattle with *Fasciola hepatica* and *Mycobacterium bovis*-immunological consequences. Transbound. Emerg. Dis.

[43] Howell A.K, McCann C.M, Wickstead F, Williams D.J L (2019). Co-infection of cattle with *Fasciola hepatica* or *F. gigantica* and *Mycobacterium bovis*:A systematic review. PLoS. One.

[44] Sabey K.A, Song S.J, Jolles A, Knight R, Ezenwa V.O (2021). Coinfection and infection duration shape how pathogens affect the African buffalo gut microbiota. ISME. J.

[45] Beechler B.R, Jolles A.E, Budischak S.A, Corstjens P.L.A, Ezenwa V.O, Smith M, Spaan R.S, van Dam G.J, Steinauer M.L (2017). Host immunity, nutrition and coinfection alter longitudinal infection patterns of schistosomes in a free-ranging African buffalo population. PLoS. Negl. Trop. Dis.

[46] Obieglo K, Feng X, Bollampalli V.P, Dellacasa-Lindberg I, Classon C, Österblad M, Helmby H, Hewitson J.P, Maizels R.M, Rothfuchs A.G, Nylén S (2016). Chronic gastrointestinal nematode infection mutes immune responses to mycobacterial infection distal to the gut. J. Immunol.

[47] Ezenwa V.O, Jolles A.E (2011). From host immunity to pathogen invasion:The effects of helminth coinfection on the dynamics of microparasites. Integr. Comp. Biol.

[48] Verhagen L.M, Hermans P.W, Warris A, de Groot R, Maes M, Villalba J.A, de Waard J.H (2012). Helminths and skewed cytokine profiles increase tuberculin skin test positivity in Warao Amerindians. Tuberculosis.

[49] Li X.X, Zhou X.N (2013). Co-infection of tuberculosis and parasitic diseases in humans:A systematic review. Parasit. Vectors.

[50] Harris N.L (2017). Recent advances in Type-2-cell-mediated immunity:Insights from helminth infection. Immunity.

[51] Kloosterman A, Frankena K, Ploeger H.W (1989). Increased establishment of lungworms (*Dictyocaulus viviparus*) in calves after previous infections with gastrointestinal nematodes (*Ostertagia ostertagi* and *Cooperia oncophora*). Vet. Parasitol.

